# Shiga Toxin Therapeutics: Beyond Neutralization

**DOI:** 10.3390/toxins9090291

**Published:** 2017-09-19

**Authors:** Gregory Hall, Shinichiro Kurosawa, Deborah J. Stearns-Kurosawa

**Affiliations:** Department of Pathology and Laboratory Medicine, Boston University School of Medicine, Boston, MA 02118, USA; grhall@bu.edu (G.H.); kurosawa@bu.edu (S.K.)

**Keywords:** Shiga toxin, Shiga-like toxins, STX1, STX2, Shiga toxin-producing *E. coli*, STEC, hemolytic uremic syndrome, unfolded protein response, ribotoxic stress response, ribotoxin

## Abstract

Ribotoxic Shiga toxins are the primary cause of hemolytic uremic syndrome (HUS) in patients infected with Shiga toxin-producing enterohemorrhagic *Escherichia coli* (STEC), a pathogen class responsible for epidemic outbreaks of gastrointestinal disease around the globe. HUS is a leading cause of pediatric renal failure in otherwise healthy children, resulting in a mortality rate of 10% and a chronic morbidity rate near 25%. There are currently no available therapeutics to prevent or treat HUS in STEC patients despite decades of work elucidating the mechanisms of Shiga toxicity in sensitive cells. The preclinical development of toxin-targeted HUS therapies has been hindered by the sporadic, geographically dispersed nature of STEC outbreaks with HUS cases and the limited financial incentive for the commercial development of therapies for an acute disease with an inconsistent patient population. The following review considers potential therapeutic targeting of the downstream cellular impacts of Shiga toxicity, which include the unfolded protein response (UPR) and the ribotoxic stress response (RSR). Outcomes of the UPR and RSR are relevant to other diseases with large global incidence and prevalence rates, thus reducing barriers to the development of commercial drugs that could improve STEC and HUS patient outcomes.

## 1. Introduction

Shiga toxins are ribotoxic proteins produced by several species of bacteria responsible for epidemic outbreaks of human gastrointestinal disease [1,2]. The prototypical toxin of this group is Shiga toxin produced by *Shigella dysenteriae* Type 1, an etiologic cause of bacterial dysentery associated with contaminated water supplies [3,4]. The related proteins Shiga-like toxin 1 (STX1) and Shiga-like toxin 2 (STX2) are produced by various pathogenic strains of Shiga toxin-producing *Escherichia coli* (STEC) responsible for food-borne illnesses globally, including numerous outbreaks in the United States, Europe, South America, and Japan [5,6,7]. STX1 and STX2 are encoded within the genome of lysogenized bacteriophages that can be transferred between related bacteria, creating a diverse array of bacterial strains secreting one or more toxin subtypes [1,8].

Shiga toxins are the etiologic cause of post-diarrheal hemolytic uremic syndrome (HUS), a thrombotic microangiopathy characterized by thrombocytopenia, hemolytic anemia, and acute renal failure following a course of bacterially induced hemorrhagic diarrhea [9,10,11,12]. Neurologic disease is a frequent complication of STEC infection via imprecisely defined mechanistic causes [12,13,14]. Approximately 5–30% of patients suffer long term morbidity from chronic renal insufficiency, hypertension, or neurological deficits following the resolution of active HUS [15]. Children younger than 2 years of age are particularly susceptible to Shiga toxin-induced HUS, and the overall HUS rates vary between 5–15% of confirmed STEC cases depending on the infecting bacterial strain. The recent European outbreak involving an atypical STEC O104:H4 strain showed substantially higher rates of adult HUS in part due to its enteroaggregative properties, and future emerging Shiga toxin-producing pathogens may have variant epidemiological profiles [6,16,17]. STEC strains are susceptible to antibiotics, but antibiotic therapy is generally contraindicated due to an association of antibiotic treatment with increased toxin production and risk of HUS development [18,19]. However, antibiotic treatment appeared to be effective during the European O104:H4 outbreak, and this was later confirmed by in vitro evaluation of patient isolates [20]. This highlights a need for rapid and specific clinical laboratory serotyping coupled with toxin detection, a technology that is not yet available commercially. As a result, the standard of care remains supportive and avoids antibiotics. The clinical management of STEC cases is complicated further by the lack of validated clinical biomarkers capable of predicting HUS onset prior to the development of thrombocytopenia and renal damage. There are no commercially approved therapeutics that specifically treat or prevent HUS caused by Shiga toxin-producing pathogens, and supportive care with careful fluid management is the recommended treatment following diagnosis [21]. Plasmapheresis and treatment with the C5 complement inhibitor Eculizumab^®^ have not shown consistent clinical benefits in human patients [22,23]. Due to the diversity of *E. coli* serotypes capable of causing Shiga toxin-mediated disease and the potential of new emerging Shiga toxin-producing pathogens, treatments that target the activity of the toxin are currently being sought to prevent the development of HUS and to improve HUS patient outcomes.

The focus of therapeutic development for Shiga toxicosis and HUS has been the blockade of toxin activity or intracellular trafficking. Thus far, no Shiga toxin-specific therapeutic has advanced past Phase II clinical trials in the United States, partially due to the difficulties in drug development for a sporadic acute disease [24]. In this review, an alternate strategy of therapeutic development is explored that proposes to target the downstream signaling and outcomes of Shiga toxin activity. The overlap of Shiga toxin-induced stress pathways with common diseases may lead to a more rapid development and approval of commercially available therapeutics to improve patient outcomes compared to the direct targeting of the toxin itself.

## 2. Shiga Toxin Structure and Activity

Shiga toxins are AB5 toxins composed of a single A subunit and a pentameric B subunit [2,25]. Shiga toxins bind to the cell membrane glycolipid globotriaocylceramide (Gb3) via three binding sites on the B subunit to initiate endocytosis and gain cellular entry [26,27]. Retrograde trafficking machinery shuttle the toxin from the early endosome through the Golgi and endoplasmic reticulum (ER) [28]. During this process, furin-like proteases cleave a target site within the A subunit to create a catalytically active A subunit [29,30]. The active toxin is then transported from the ER into the cytosol to reach its target, the 28S rRNA of ribosomes [31,32]. Shiga toxins depurinate the conserved adenine residue 2260 within the sarcin–ricin loop of 28S eukaryotic rRNA via N-glycosidase activity to inhibit binding by the elongation factor EIF2a, thus terminating peptide elongation [31,33]. Other ribosome-inactivating toxins act on the sarcin–ricin loop of 28S eukaryotic rRNA, and the specific mechanism of toxicity via depurination is shared between Shiga toxins and the plant-derived toxin ricin [32]. Shiga toxins are highly toxic to Gb3-positive cells in culture, though their sensitivity is known to vary widely between cell lines and toxin subtypes [34,35,36]. Shiga toxicity in rodents, pigs, rabbits, and non-human primates differs in the distribution of tissues affected, presumably due to species differences in cellular Gb3 expression and localization. Rodents and rabbits develop gastrointestinal and renal tubular epithelial lesions when challenged with toxin, but fail to develop the glomerular endothelial damage and clinical HUS seen in intoxicated human patients and non-human primates [37,38,39,40]. Toxin-induced alterations in ribosomal structure and activity initiate cell stress pathways known as the unfolded protein response (UPR) and the ribotoxic stress response (RSR), which activate a variety of pro-inflammatory and pro-apoptotic cellular effector proteins that contribute to cellular dysfunction and disease [41,42,43,44,45,46]. The outcomes of these stress responses are inflammatory cytokine secretion, cellular apoptosis, and endothelial dysfunction, all of which are potential contributors to Shiga toxin-induced disease in vivo.

## 3. Current State of Shiga Toxin-Targeted Therapeutic Development

**Figure 1:**
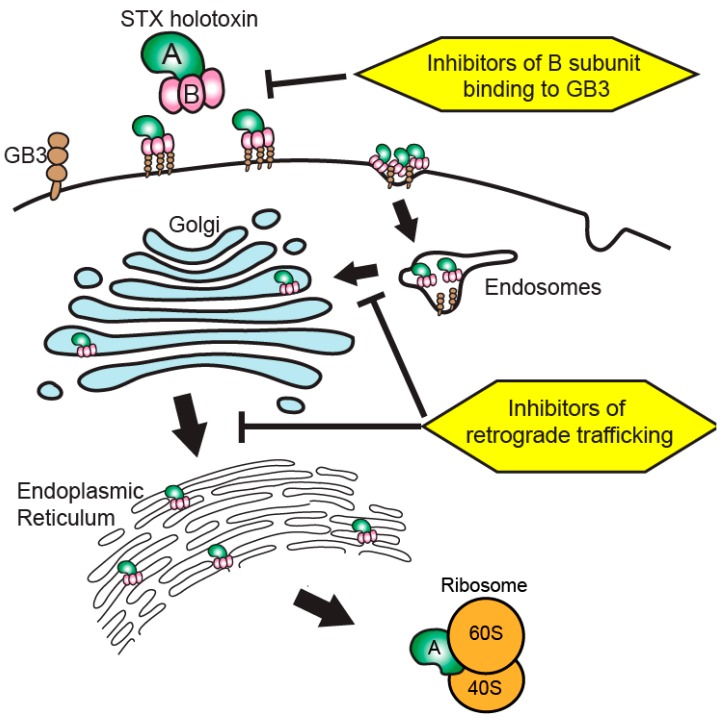
**Therapeutic targeting of Shiga toxin internalization and retrograde trafficking.** Shiga toxins bind to cell membrane globotriaosylceramide (Gb3) to initiate internalization via endocytosis. The toxin then undergoes retrograde trafficking from endosomes through the Golgi apparatus and endoplasmic reticulum. The holotoxin is processed during trafficking to release active toxin A subunits into the cytosol to interact with 60S ribosomal components, resulting in ribotoxicity through depurination of rRNA adenine residues within the sarcin–ricin loop. Therapies inhibiting the interaction of Shiga toxin B subunits with Gb3 at the cell surface or inhibiting aspects of the retrograde trafficking system seek to ameliorate Shiga toxicity by preventing the toxin from reaching cytosolic ribosomes to initiate ribotoxicity. STX: Shiga-like toxin.

The field of Shiga toxin therapeutic development has focused on inhibiting the toxins at various points in the pathway between entry into the cell and ribosomal depurination by activated toxin A subunits (Figure 1). The therapeutic targeting of the toxin is conceptually justified due to a direct causal link between Shiga toxin and the development of HUS [10,12]. While this review will briefly summarize the current state of toxin-directed therapeutics, a more complete review was recently provided by Melton-Celsa and O’Brien [24].

Toxin-neutralizing therapeutics and vaccines for other toxin-based diseases, such as tetanus and anthrax, have yielded excellent clinical outcomes, thus providing inspiration for Shiga-like toxin (STX)-neutralizing therapeutic strategies [47,48] (Table 1). Trials using polyclonal anti-sera or monoclonal anti-Shiga toxin antibodies in animal models of Shiga toxicosis successfully rescue from mortality if treatment is given within 48 h of toxin exposure [49,50,51,52]. Monoclonal humanized murine anti-Shiga toxin antibodies have reached Phase II clinical trials within the U.S., but none have completed Phase III trials [53,54]. Camelid heavy-chain-only antibody constructs containing heavy-chain oligomers specific for the toxin have been developed and are effective in murine and porcine models, but have not been used in humans to date [55]. The heavy-chain antibody constructs have been administered as a bolus injection or introduced via a replication-incompetent adenoviral construct, with both routes of administration conferring protection against STXs [56]. One of the challenges facing antibody-based Shiga toxin neutralizers is the variety of toxin subtypes present in various STEC strains. A monoclonal antibody must neutralize STX1 and multiple STX2 subtypes to be universally effective in STEC patients, since each STEC strain can secrete one or more subtypes.

Specific multivalent binding of the Shiga toxin B subunit to Gb3 in all STX subtypes relevant to the human disease has led to the generation of synthetic Gb3 analog constructs capable of inhibitory toxin binding. Synsorb-Pk^®^ is a silicon dioxide backbone containing bound Gb3 capable of neutralizing Shiga toxins in vitro [57]. A Phase II trial in pediatric HUS patients failed to show any clinical improvement following treatment with Synsorb-Pk^®^, possibly due to the inability of the orally administered drug to adsorb toxin produced at the mucosal surface or injected directly into epithelial cells by STEC [58]. The Nishikawa group has generated metallic backbone tetravalent peptides capable of binding to the Gb3 binding sites of STX1 and STX2 B subunits [59,60]. Variations of the construct have been found to be protective when administered systemically in murine and non-human primate models of Shiga toxicosis, but no human trials have been performed [61,62]. It remains to be seen if immunologic or Gb3-analog toxin neutralizers can be effective in the clinical environment where STEC-infected patients have likely been exposed to Shiga toxins for several days prior to presentation, or if preventing further toxin internalization will improve patient outcomes.

The blockade or alteration of the retrograde trafficking system is an alternate strategy for the inhibition of Shiga toxin activity. Stechmann et al. characterized two small molecule re-localizers of the vesicular transport SNARE protein Syntaxin 5 that were capable of rescuing protein synthesis in cells incubated with ricin and Shiga toxins for 4 h [63]. They named the compounds Retro-1 and 2, and found that the drugs did not impact the localization of several other cargo transport proteins in vitro. Retro-2 was found to be partially protective in a murine model of STEC infection when given orally prior to bacterial toxin induction with mitomycin C and completely protective against nasal ricin challenge if given prophylactically [63,64]. The metallic cofactor manganese has also been found to protect mice from STX1 toxicity by stimulating the degradation of the endosome-to-Golgi transport protein Gpp130, but doses were given prophylactically every 24 h for 5 days prior to a single bolus injection of toxin [65]. Manganese failed to protect mice from STX2 toxicity due to STX2 transport through proteins other than Gpp130, limiting its usefulness clinically [66]. To date, there have been no published accounts of reduced clinical toxicity when retrograde trafficking inhibitors are given after toxin exposure in vivo, a condition that more closely replicates the likely clinical scenario in diagnosed STEC patients.

## 4. Beyond Toxin Neutralization

While many toxin neutralizers and inhibitors have shown promise in pre-clinical settings and limited Phase II clinical trials, a direct inhibitor of Shiga toxins has not been successfully brought to market to prevent or ameliorate HUS in clinical STEC patients. Commercial barriers to treatments that specifically target sporadic acute epidemic diseases include difficulties in distribution and storage of the therapeutic as well as a reduced financial incentive to develop and produce therapeutics for an inconstant patient cohort. In addition, the usual HUS rate during an outbreak makes the statistics and finances for a clinical trial almost insurmountable. A simple design of two study groups (STEC versus STEC + HUS; α = 0.05, power 80%) and a 5% HUS rate will need over 600 patients to show effective drug prevention of HUS, which has been the criteria required by the FDA. Most outbreaks are less than 100 patients [5,72]. It is with these barriers in mind that an alternate strategy for STEC and HUS therapy should be considered that does not directly inhibit toxin activity but instead attempts to modulate the downstream stress responses to the catalytic activity of the toxin. Shiga toxins induce cellular stress pathways in sensitive cells following ribotoxicity and translational inhibition. Unfolded or incomplete proteins are detected in the ER by sensor proteins to initiate signaling cascades termed the unfolded protein response (UPR), and changes in ribosome conformation caused by depurination of the sarcin–ricin loop initiate separate signaling pathways termed the ribotoxic stress response (RSR). The UPR and RSR are relevant to other diseases, thus increasing the number of commercially viable drug candidates for the treatment of STEC and HUS patients. It is also possible that by the time of diagnosis, Shiga toxin internalization, processing, and ribosomal inactivation have already progressed to the point where toxin neutralizers and inhibitors alone will not be the most effective way of reducing cellular damage and HUS development. Targeting the UPR and RSR in STEC patients may be successful clinically, either alone or in combination with anti-toxin therapy.

## 5. The Unfolded Protein Response (UPR)

The UPR is a eukaryotic cellular stress response triggered by the accumulation of unfolded or improperly folded peptides within the lumen of ER, also known as ER stress. The UPR is initiated by several proteins located within the ER lumen that sense unfolded proteins and are then activated to initiate signaling cascades that attempt to restore protein homeostasis. The best characterized sensors of ER stress are protein kinase R-like endoplasmic reticulum kinase (PERK), inositol-requiring protein 1α (IRE1α), and activating transcription factor 6α (ATF6α) [73].

IRE1α is an ER membrane-bound protein that activates its endoribonuclease activity via the dissociation of the inhibitory chaperone protein GRP78 during ER stress. IRE1α cleaves a 26 nucleotide segment from xbox binding protein 1 (XBP1) mRNA. The frameshift created by this cleavage leads to the translation of the active form of XBP1, which acts as a transcription factor for protein folding, protein transport, protein degradation, and mRNA degradation-associated genes. Concurrent with IRE1α activation, the ER stress sensor protein ATF6α moves from the ER to the Golgi for processing into its active form. Active ATF6α travels to the nucleus to upregulate the transcription of chaperone proteins. The combination of ATF6α and IRE1α activation leads to an upregulation of protein-folding and degradation machinery to restore protein homeostasis [73,74].

The ER membrane-bound kinase PERK is activated to initiate a separate signaling pathway during ER stress via phosphorylation and the inactivation of eukaryotic translation initiation factor 2a (eIF2a). The inactivation of eIF2a inhibits translation to allow cellular machinery time to properly fold or degrade misfolded proteins. Phosphorylated eIF2a simultaneously upregulates several transcripts coding for proteins involved in protein degradation, mRNA degradation, chaperone proteins to aid in peptide folding, and protein transporters. A key known transcriptional target of phosphorylated eIF2a is activating transcription factor 4 (ATF4). ATF4 is a transcription factor for antioxidant and amino acid biosynthesis genes as well as CCAAT/enhancer-binding protein (C/EBP) homologous protein (CHOP). CHOP forms heterodimers with ATF4 to upregulate the transcription of additional UPR targets, including growth arrest and DNA-damage-inducible protein 34 (GADD34). GADD34 is a phosphatase that dephosphorylates eIF2a to reinitiate ribosomal translation [73,74,75]. The activation of ATF6α and IRE1α occurs transiently during ER stress, but the activation of PERK persists until ER stress is resolved [76].

While a short term activation of the UPR promotes survival of the cell, chronic UPR activation eventually leads to cellular apoptosis. The transcription factor CHOP concurrently suppresses anti-apoptotic Bcl-2 expression and enhances pro-apoptotic factor expression if chronically present in the cell. CHOP activates ER oxidase 1α (ERO1α), a protein that facilitates disulfide bond formation in the ER [77]. In the context of unresolved ER stress, the reactive oxygen species generated by disulfide bond formation lead to increased Ca^+2^ efflux from the ER, which in turn causes mitochondrial stress. Resuming ribosomal translation via GADD34 phosphatase activity on eIF2a in the context of unresolved ER stress leads to further generation of reactive oxygen species and ATP depletion [75]. CHOP appears to be the key factor in the progression of the UPR to apoptosis, as the knockout of CHOP in cultured cells and mice protects from apoptosis in various models of chronic UPR [77,78]. A chronic upregulation of CHOP leads to an upregulation of Bcl-2 interacting mediator of cell death (BIM) protein expression with a concurrent downregulation of the anti-apoptotic protein Bcl-2. BIM activates caspase 3, which in turn activates the caspase 8 executioner complex to initiate apoptosis [73].

## 6. The UPR in Health, Disease, and Shiga Toxicosis

Although the UPR is considered a stress response, it is a critical process involved in the normal function of eukaryotic organisms during development, cellular differentiation, and during times of intense cellular metabolism. During the differentiation of cells, large scale shifts in protein synthesis occur that can transiently lead to excess amounts of unfolded proteins. B cell development is a known example where abrogation of the UPR hinders cellular functional differentiation [79,80,81]. Cells that acutely produce and secrete proteins in response to stimulus also rely on the UPR to function normally and avoid cell death. Insulin-producing beta cells of the pancreas and activated inflammatory cells secreting cytokine and chemokine proteins are known to activate the UPR during stimulation [82,83,84].

Induction of the UPR by Shiga toxins has been documented in various susceptible cell types in culture as well as the renal tissue of in vivo mouse models of Shiga intoxication. While apoptosis occurs in susceptible cells exposed to Shiga toxins, it remains unclear if the UPR is the key driver of cellular apoptosis or if other cell stress response pathways, such as the RSR and TNFα death signaling, are involved [85,86]. STXs are known to associate with the ER chaperone proteins HEDJ and BiP during retrograde transport, possibly contributing to initiation of the UPR in addition to ribosomal inactivation [87,88]. Human monocytic leukemia cells upregulate IRE1α, p-PERK, and CHOP when exposed to STX1, and subsequently generate active forms of XBP1 and caspase 8 [44]. The knockdown of CHOP in THP-1 cells prevents apoptosis and caspase 8 activation [89]. Human brain microvascular endothelial cells showed a similar upregulation of CHOP and generation of caspase 8 when exposed to STX2 [90]. Mouse models injected with purified STX2 or infected with the intestinal pathogen *Citrobacter rodentium* carrying a STX2-containing plasmid showed increased renal expression of CHOP and spliced XBP1 transcripts as well as reduced Bcl-2 transcripts [46].

The UPR is also induced in a variety of diseases associated with chronically increased cellular protein production and/or secretion (Table 2). Insulin resistance and diabetes chronically induce the UPR in insulin-producing beta cells, leading to beta cell loss over time [84,91]. In diabetes-prone mice, the deletion of CHOP and other late-UPR factors rescues mice from the loss of beta cells. Non-alcoholic fatty liver disease leads to chronic UPR in hepatocytes due to increased fatty acid metabolic pathway activity, eventually contributing to intracellular lipid accumulation and hepatocyte dysfunction [92]. Neurons upregulate the UPR during neurodegenerative diseases associated with protein aggregates, such as Parkinson’s disease and Alzheimer’s disease [93]. Defective UPR responses in gastrointestinal Paneth cells have been suggested to contribute to inflammatory bowel disease, with unresolved ER stress leading to inflammation and a loss of microbiome homeostasis [94,95]. ER stress responses have also been found to contribute to cardiac dysfunction following hypoxia, and are associated with aberrant angiogenesis during vascular retinopathies and neoplastic growth [96,97,98,99,100]. Therapeutic development targeting the UPR is relevant for common chronic diseases, and could also be promising for the treatment of Shiga-intoxicated patients, thus reducing economic barriers for the development of treatments for STEC and HUS.

## 7. Targeting the UPR

**Figure 2:**
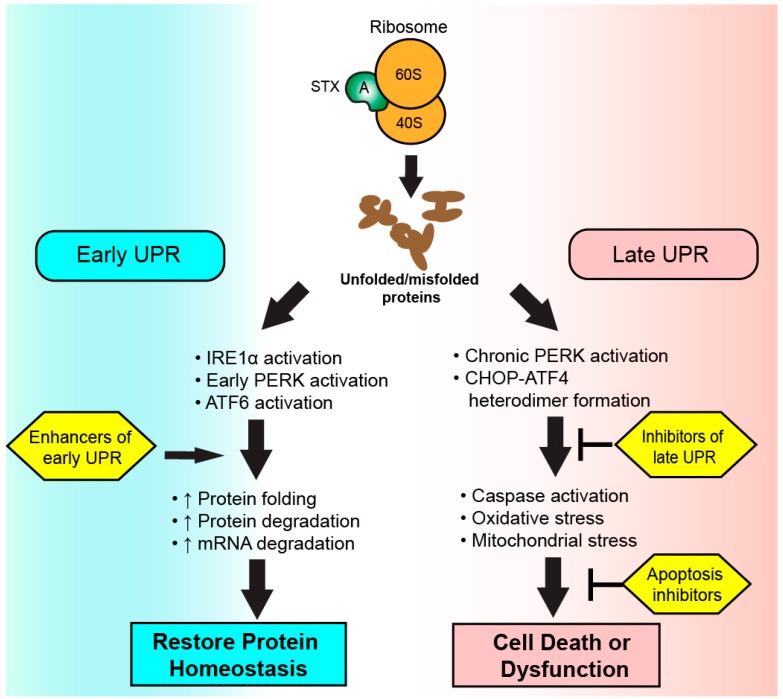
**Therapeutic targeting of the unfolded protein response (UPR) during Shiga toxicity.** Following ribosomal inhibition by Shiga toxin A subunits, an accumulation of unfolded and misfolded proteins is detected by sensor proteins to initiate the UPR. Therapeutics enhancing the early UPR seek to increase cellular capacity to resolve endoplasmic reticulum stress via the restoration of protein homeostasis. A chronic activation of UPR results in apoptosis or cellular dysfunction via the activity of CHOP-ATF4 heterodimers. Therapies targeting the late UPR seek to inhibit the activity or formation of CHOP–ATF4 heterodimers or inhibit initiators of apoptosis to preserve cellular function.

The most promising approach to the treatment of UPR-related diseases is to target the downstream effectors of chronic CHOP–ATF6 activity leading to the upregulation of apoptotic factors, the downregulation of anti-apoptotic factors, and the eventual activation of caspase 3 and caspase 8 to initiate apoptosis (Figure 2). A high-throughput therapeutic screening technique validated this theory in a variety of bacterial toxins including ricin, anthrax toxin, and diphtheria toxin by identifying a universally protective compound called biothionol. Biothionol is a small molecule caspase 3, 6, and 7 inhibitor found to prevent cytotoxicity by ricin in RAW264.7 and C32 cells in the face of ribosomal inactivation, and did not show signs of toxicity in mice [101]. Bithionol has not been used in STX models to date. Ouabain is a cardiotonic steroid that decreases Ca^+2^ fluxes intracellularly and increases Bcl-XL expression via the activation of Nf-KB p65 subunits, thus blunting the outcomes of chronic UPR activation. Treatment of rat proximal renal tubular cells exposed to STX2 with Ouabain in vitro prevented caspase 3 activation and cellular apoptosis. Ouabain was also found to protect human renal tubular epithelial cells and human glomerular endothelial cells from STX2-induced apoptosis in vitro [101]. Treatment of mice with continuous subcutaneous infusion of Ouabain prevented renal tubular epithelial apoptosis and a loss of podocytes 48 h after injection with STX2.

An alternate protective strategy is to increase the cellular capacity to resolve the UPR in an attempt to avoid chronic ER stress (Figure 2). Small molecule protein chaperones that improve protein folding in cells undergoing ER stress improve outcomes in mouse models of diabetes and inflammatory bowel disease, and have shown positive results in human patients with insulin resistance or cirrhosis [102,103,104,105]. Extendin-4 is an agonist of glucagon-like peptide agonist 1 that increases the expression of ATF4 in pancreatic beta cells to increase the protective effects of the early UPR [106]. Extendin-4 has been approved for use in human diabetics in the United States and Europe. To date, therapeutics that enhance UPR resolution have not been studied in the context of Shiga toxin-mediated disease in vitro or in vivo.

## 8. The Ribotoxic Response

In addition to the UPR, damage to domains V or VI of the ribosomal 28S rRNA has been found to initiate a stress response signaling cascade termed the ribotoxic stress response (RSR) [107]. The activity of ribotoxins such as ricin, sarcin, anisomycin, and deoxynivalenol (DON) leads to the activation of double-stranded-RNA-activated kinase R (PKR), likely through the homodimerization and transphosphorylation of ribosome-associated PKR [108]. PKR phosphorylates the translation factor eIF2a to inactivate it and initiate a signaling cascade leading to variable activation of classical p38 MAP kinases, ERK, and JNK depending on the cell type. It should be noted that both the UPR and the ribotoxic response involve the phosphorylation of eIF2a, but in cell culture experiments the ribotoxic stress response could be elicited at low levels of translational inhibition by ribotoxins. Furthermore, ribotoxins, such as emetine and pactamycin, that act on ribosomal regions other than domain V or VI fail to elicit the activation of PKR with downstream kinase activation despite their ability to inhibit protein synthesis [107]. These findings suggest that the RSR is an independent stress pathway that is distinct from the UPR and is not reliant on translational inhibition. Hematopoetic cell kinase (Hck) was found to associate with PKR and the 40S ribosome in human monocytic U937 cells, and the pharmacological inhibition of Hck activity prevented p38 MAPK phosphorylation and IL-8 secretion in response to DON, suggesting that Hck is also a key sensor of ribosomal damage necessary for downstream effector kinase signaling [109]. While the details of the signaling pathway between PKR and downstream effector kinase activity remain unclear, the zipper sterile alpha motif kinase (ZAK) has been identified as a critical component to signaling via pharmacological and siRNA knockdown studies in vitro. An abrogation of ZAK activity prevents the activation of p38 MAPK, JNK, and ERK in Hct-8 and Vero cells incubated with STX1 [81]. The ribotoxic stress response may also lead to cell death if chronic stimulation occurs, with NLRP3 inflammasome activation and pyroptosis via caspase 1 activation documented in THP-1 cells incubated with active STX1 in a concentration-dependent manner [41].

The in vitro impact of the RSR varies depending on the cell type, but is generally characterized by an upregulation of inflammatory cytokine and chemokine transcription and translation. Differentiated macrophage-like THP-1 cells secrete TNFα, IL-1β, IL-8, and MIP-1α in response to stimulation with STXs, and the response could be blunted through an inhibition of JNK, p38 MAPK, and ERK with variance in effect based on the kinase inhibited [43,110,111,112]. Human intestinal epithelial Hct-8 cells secreted the chemokine IL-8 when incubated with STX1 or the ribotoxic antibiotic anisomycin, and concurrent incubation with *E. coli* flagella and STX2 led to a superinduction of IL-8 secretion compared to flagella alone [113,114]. Interleukin 8 secretion was reduced through the inhibition of ERK, p38 MAPK, or JNK signaling or through use of a ZAK inhibitor. The amplification of MAPK, JNK, and ERK phosphorylation has also been documented in human dendritic cells co-stimulated with lipopolysaccharide (LPS) and anisomycin, with increased TNFα and IL12 secretion compared to stimulation with LPS alone [115]. Human brain endothelial cells secreted IL-6 and IL-8 in response to STX1, and the amount of protein secreted was amplified by costimulation with TNFα and STX1 concurrently [116,117]. Human microvascular endothelial cells were found to upregulate the chemokines IL-8, CXCL4, CXCR7, and CXCL12 in response to challenge with STX holotoxins, with delayed degradation of chemokine transcripts [118].

In vivo experiments in mice and non-human primates (NHPs) have documented acute tissue inflammation in the kidneys as well as increased circulating acute inflammatory proteins following exposure to Shiga toxins via injection or gastrointestinal infection with STEC [40,52,119]. In both mice and NHPs, increased circulating and renal TNFα, IL-6, IL-1β, and CXC chemokines are present following injection with purified STX1 and STX2. Mice lacking the gene encoding ZAK were protected from gastrointestinal ricin toxicity, with reduced CXCL1 production following depurination of the sarcin–ricin loop [120]. The treatment of rabbits with the ZAK kinase inhibitor imatinib reduced the number of neutrophils infiltrating colonic tissue infected with STEC [121]. In human HUS patients, serum and urine IL-6 levels correlated with severity of disease, and HUS patients suffering from neurologic complications had detectable increases in brain IL-1β content [122,123,124].

## 9. Targeting the RSR and Inflammation during Shiga Toxicosis

Limited research has been performed to evaluate the impact of targeted anti-inflammatory therapy on susceptibility to STX-mediated tissue injury, morbidity, and mortality. Alves-Rosa et al. hypothesized that the enhanced inflammatory response documented in vitro in cells costimulated with STXs and LPS could be blunted with anti-LPS antibodies in vivo. Mice immunized with *E. coli* O111:B4 were challenged with LPS and STXs following the confirmation of circulating anti-LPS IgG antibodies. There was no difference in mortality in immunized mice following STX + LPS challenge; however, the immunized mice failed to suppress circulating TNFα following intravenous LPS challenge compared to naïve mice, suggesting that the immunization did not prevent pattern recognition receptor activation by circulating LPS [125]. Mice pretreated with immunosuppressive doses of dexamethasone exhibited greater survival following STX2 challenge, reduced numbers of Gb3-positive CNS cells, reduced damage to the blood brain barrier, and a reduction in the number of activated astrocytes compared to controls [126]. The vasoactive drug anisodamine, an inhibitor of TNFα secretion, improved the survival of mice injected with lethal doses of STX. The improved survival was reversed via a concurrent injection of recombinant TNFα with STX, suggesting that the suppression of TNFα by anisodamine provided significant protection from STX-induced disease [127]. The pretreatment of mice with MCP-1-, MIP-1α-, or RANTES-neutralizing antibodies protected against renal fibrin deposition following STX + LPS injections [128]. Isogai et al. found that germ-free mice pretreated with anti-TNF antibodies and infected with STEC were protected from clinical signs of morbidity, reduced renal pathology on histology, and reduced renal IL-1β, TNFα, and IL-6 protein concentrations despite similar levels of intestinal colonization and fecal STX content compared to controls [129].

**Figure 3:**
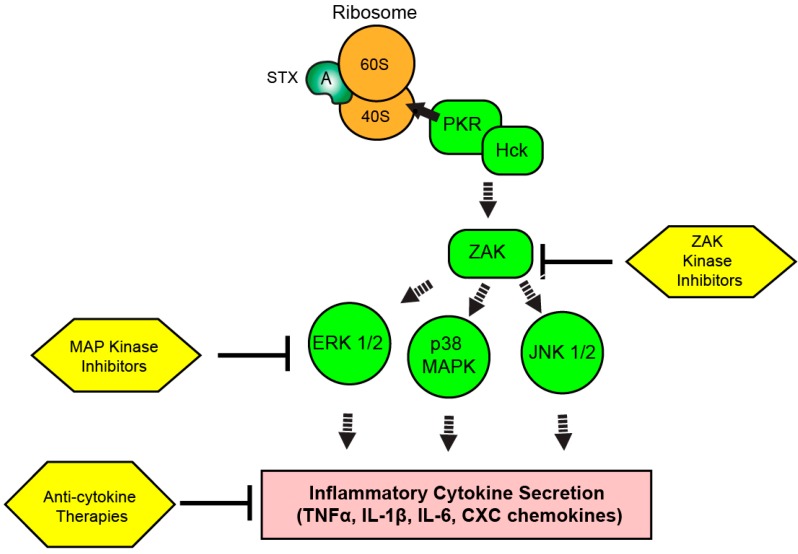
**Therapeutic targeting of the ribotoxic stress response (RSR) during Shiga toxicity.** Depurinated ribosomes are detected by PKR and Hck complexes to initiate the RSR. Signaling cascades involving ZAK and MAP kinases result in the upregulation of inflammatory transcripts and cytokine secretion depending on the intoxicated cell type. Kinase inhibitors targeting the RSR pathway or inhibitors of secreted cytokine activity could modulate Shiga toxicity through reduction of inflammatory cytokine-driven components of disease progression.

A broad range of diseases are either driven through inflammatory responses or are complicated by inflammatory dysfunction. Anti-cytokine and chemokine therapeutics are being developed for use in diseases ranging from rheumatoid arthritis to inflammatory bowel disease, with multiple commercially approved drugs already available in the United States [130,131,132,133]. General anti-inflammatory drugs, such as glucocorticoids and NSAIDs, have been available for decades to treat acute inflammatory diseases. Further preclinical studies are necessary to characterize the role of inflammation in the pathogenesis of STX-induced HUS, as the role of the host response to STEC infection in the pathogenesis of HUS remains unclear [10,11,12]. Further work is necessary to determine if specific host cytokine responses are necessary for the development of HUS to determine the suitability of RSR elements as therapeutic targets for clinical study (Figure 3).

## 10. Future Directions

Research focusing on traditional toxin neutralizers has yielded several candidate molecules successful in animal models, but none have achieved FDA approval for use in patients. The epidemiologic profile of STEC outbreaks complicates pharmaceutical development due to its acute, sporadic, and geographically dispersed distribution of cases. Focusing on the downstream molecular impacts of STX, such as the UPR and RSR, have the advantage of utilizing therapeutics developed for use in common chronic diseases that have a greater financial incentive for pharmaceutical development. Further preclinical study is required to determine the roles of the UPR and RSR during development of HUS in order to identify and validate potential novel therapeutic targets to improve STEC and HUS patient outcomes.

## Figures and Tables

**Table 1:** Shiga toxin-directed therapeutics in pre-clinical and early clinical development.

Therapeutic	Drug Class	Target	Mechanism of Action	Animal Models Tested	Clinical Trials Completed	References
Anti-sera	Polyclonal antibodies	STX, STX2	Circulating toxin neutralization	Pig, rabbit	None	[49,67]
Urtoxezumab^®^	Humanized murine monoclonal antibody	STX2	Circulating toxin neutralization	Rodent, NHP	Phase II	[50,53]
cαSTX1 and cαSTX2	Humanized murine monoclonal antibody	STX1, STX2	Circulating toxin neutralization	Rodent	Phase I	[54,68]
Murine anti-STX2	Murine monoclonal antibody	STX2	Circulating toxin neutralization	Rodent	None	[52]
Anti-STX antibodies (various clones)	Human monoclonal antibody	STX1, STX2	Circulating toxin neutralization	Rodent, pig	None	[51,69,70]
Camelid anti-STX oligomers	VHH-based neutralizing agent	STX1, STX2	Circulating toxin neutralization	Rodent	None	[55,71]
Adenoviral anti-STX2 construct	VHH-based neutralizing agent	STX2	Circulating toxin neutralization	Rodent, pig	None	[56]
Tetravalent peptides	Gb3 analogs	STX1, STX2	Circulating toxin neutralization	Rodent, non-human primate	None	[59,60,61,62]
Synsorb-Pk^®^	Silicon dioxide-Gb3 construct	STX1, STX2	Gastrointestinal toxin neutralization	None	Phase II (failed)	[57,58]
Retro 1 and Retro 2	Small molecule inhibitors	STX1, STX2	Retrograde trafficking inhibitor	Rodent	None	[63,64]
Manganese	Enzyme cofactor	STX1	Retrograde trafficking inhibitor	Rodent	None	[65]

**Table 2:** Diseases associated with Unfolded Protein Response (UPR) Activation.

Disease	Cells Type Affected	Outcome	Model System(s)	Reference
Shiga toxicosis following STEC infection	Leukocytes, endothelial cells, renal epithelium, gastrointestinal epithelium	Hemolytic uremic syndrome? Inflammatory cytokine secretion?	Rodent, human monocyte, renal epithelial, and endothelial cells in vitro	[34,44,46,89,90]
Diabetes mellitus	Pancreatic beta cells	Loss of insulin production	Rodent	[91,92]
Obesity	Hepatocytes	Hepatic lipidosis, insulin resistance	Rodent, various hepatocyte cell lines in vitro	[91,92]
Inflammatory Bowel Disease	Intestinal Paneth and goblet cells	Loss of Paneth cells, gastrointestinal inflammation	Rodent	[94,95]
Neurodegenerative Diseases	Neurons	Neuron dysfunction and degeneration	Rodent	[93]
Vascular retinopathies	Retinal endothelial and pigmented epithelial cells	Aberrant angiogenesis	Rodent, human retinal endothelial cells and pigmented retinal epithelial cells in vitro	[96]
Cardiac disease	Cardiomyocytes	Cardiac hypertrophy, arrhythmias, cardiac fibrosis	Rodent, rabbit, human cardiomyocytes in vitro	[97]
Neoplasia	Malignant cells	Inflammatory cytokine secretion, angiogenesis, tumor survival	Human-mouse xenografts, neoplastic cells in vitro	[98,99,100]

STEC: Shiga toxin-producing enterohemorrhagic *Escherichia coli*.
